# National Governance of Public Health Responses in a Pandemic?

**DOI:** 10.1017/err.2020.39

**Published:** 2020-04-21

**Authors:** Mary DOBBS

**Affiliations:** * Law lecturer at Queen’s University Belfast, UK; email: m.dobbs@qub.ac.uk.

## Abstract

The world is currently facing the worst pandemic in a century and we were caught unprepared. COVID-19 has proven highly contagious and with severe consequences that are still unfolding. As of 16 April 2020, there were over 2 million *confirmed* cases and over 136,000 related deaths reported worldwide.^1^ Over 1 million of those confirmed cases were in the preceding 14 days, with the USA accounting for nearly half of those. Furthermore, the International Monetary Fund (IMF) is now warning that the world is about to suffer the worst economic recession since the Great Depression in the 1920s.^2^

The world is currently facing the worst pandemic in a century and we were caught unprepared. COVID-19 has proven highly contagious and with severe consequences that are still unfolding. As of 16 April 2020, there were over 2 million *confirmed* cases and over 136,000 related deaths reported worldwide.^1^ Over 1 million of those confirmed cases were in the preceding 14 days, with the USA accounting for nearly half of those. Furthermore, the International Monetary Fund (IMF) is now warning that the world is about to suffer the worst economic recession since the Great Depression in the 1920s.^2^

However, although the World Health Organization (WHO) provides scientific expertise globally and some other examples of limited centralisation exist (eg the European Union (EU) provides for minimum quality standards regarding medical products or food), public health is primarily governed at a national level or regional level (within the nation state). Consequently, despite some overlap in mechanisms such as contact tracing and social distancing, responses have varied considerably in objectives, timing and degree – even within the EU or across the USA.

This raises the fundamental question of whether national decision-making is effective or indeed appropriate in the context of the COVID-19 or similar future pandemics,^3^ or whether a supranational or international approach would be more appropriate. In order to address this question, the nature of COVID-19 and the policy responses are analysed through the lens of subsidiarity.

## I. Subsidiarity-based multilevel governance^4^

Whilst the nation state and Westphalian sovereignty remain the starting points when considering regulatory powers within a territory and engagement on the international sphere, these are not set in stone and considerable variations arise. Thus, multilevel governance and regulation theories acknowledge the “reallocation of authority upwards, downwards and sideways from central states”.^5^ This begs the question of how to determine where the core powers *ought* to rest.

One potential mechanism is by applying subsidiarity – a broad concept with roots in ideas of democracy, Catholicism and economics or effectiveness.^6^ It focuses on “the proper geographic distribution of power”.^7^ This broadly argues that powers ought to rest at the lowest level possible (due to democracy), unless it would be more effective to allocate them at a higher level.^8^ There are three key steps in order to apply subsidiarity, with a range of considerations within them.^9^

The first relates to the interest(s) in question. It is necessary to identify them and consider how significant they are to the various levels or constituents, to what extent homogeneity or heterogeneity exists (eg regarding objectives, balance with other interests and broad approaches) and the capacity of other levels to accommodate the heterogeneity. In the context of public health, this normally includes considering issues such as whether there is broad consensus on acting as a welfare state or not and the balance with other societal issues where resources are insufficient, as well as opinions on related issues such as the approach to the economy and markets. Whilst each state shares values and goals of strong public health and also a resilient economy, with both closely intertwined in the long term, there is clearly no broad global consensus on the balance between values and approaches to them.

The second entails considering the question of effectiveness or efficiency. This includes identifying where the relevant expertise and/or knowledge lie, including whether there is access to resources at a different level or not. In many contexts, this may include local and experiential knowledge. Where scientific or other expertise is central to decision-making, centralisation of both the research and the decision-making may be efficient, as lower levels may not have the necessary resources and “gaps” could arise.^10^ However, in cases of uncertainty, the value of full centralisation may be more questionable. It also includes identifying the potential for externalities, whereby one body’s decisions can impact on external bodies and viceversa, and the potential to internalise those externalities or not by centralising. Where there are significant negative externalities, this would support centralisation.

Then finally a balancing act must be undertaken – making for a very complex calculation, where the division and (re-)allocation of different powers across several levels may be appropriate.

But how does this then apply to COVID-19 and the surrounding decision-making?

## II. COVID-19

Whilst still part of public health, COVID-19 goes beyond the norm. Firstly, COVID-19 is highly contagious and spreads swiftly and easily.^11^ This is accentuated by potentially long incubation periods and the potential for an individual to be contagious even though they are asymptomatic. The virus also remains viable on surfaces such as paper and plastic for some time, facilitating the spread further. Without control measures, the spread has proven to be exponential – with us all now becoming too familiar with steeply increasing graphs and the term “R0”.^12^ Nor is this limited to one area or country. Due to a combination of the above factors, in conjunction with the widespread travel of individuals and products, the virus has spread globally and become a pandemic.

Furthermore, COVID-19 has devastating effects. We have no vaccines, no tailored effective treatments and no built-in resistance or partial immunity to COVID-19. Whilst most will be asymptomatic or have mild symptoms, it can be fatal, with the elderly, ill and those with “underlying conditions”^13^ being particularly vulnerable, and it also can cause severe impairments in the short and long term – although the extent of these impairments is still to be fully understood.^14^ This has further knock-on effects, as some public health systems are overburdened to the point of collapse – illnesses or injuries that should be manageable cannot be properly treated, appointments and treatments are postponed and discussions arise about triage for both COVID-19 and non-COVID-19 patients.^15^

The combination of the highly contagious nature and severity of impacts is worrying. It also provides for clusters of cases affecting specific groups and industries, including both healthcare workers (exacerbating the public health impacts once more) and other “essential workers” in global supply chains,^16^ as well as the economy reflected in the IMF’s report. All of these feed into each other and risk circular short- and long-term effects – where the virus is present and elsewhere. Consequently, there is the potential for severe externalities by the virus itself if left unmanaged – but this does not mean that the governance of it would lead necessarily to externalities also.

But is effective governance possible? A final major concern for the moment, which impacts upon every nation as they react to this, is the underlying uncertainties.^17^ We do not understand the disease fully, gathering data and hopefully understanding as we go. That uncertainty relates to when an individual is contagious, the morbidity levels, how to test for the virus, how to test for antibodies, how long a person might be immune for and how to treat or prevent it in future. Whilst major strides are being made with each of these aspects, uncertainty still remains – in particular regarding antibodies, immunity and vaccines. Furthermore, testing (and subsequent contact tracing) for the disease is sometimes flawed and frequently limited – hampering understanding and making for patchy data to underpin decision-making. Consequently, any decision-making is based on the precautionary principle (whether express or otherwise) and it becomes more challenging to identify clear pathways to address the pandemic effectively that also minimise countervailing risks – something that may in itself justify national rather than international approaches, even whilst benefiting from the centralisation and sharing of scientific data.

## III. Pandemics policies?

In light of the goal of public health, the ideal options would be widespread immunity of the entire population and/or complete eradication of the virus, with effective treatment as a support mechanism. However, besides the surrounding uncertainty, nation states have different capacities, and any response has countervailing risks. Consequently, nation states have chosen different approaches.

The core responses include herd immunity,^18^ “flattening the curve”^19^ and complete eradication.^20^

(1) Herd immunity is where a sufficient proportion of the population have immunity to a disease so that no further members of the population are likely to be affected in the future. For diseases such as polio, this is largely achieved through vaccination, but as there is no current vaccination for COVID-19, to achieve this now would entail initially enabling the spread of a disease through a population (as with chicken pox parties).
(2) “Flattening the curve” involves trying to control the spread of the disease so that the increase in cases, especially those requiring medical treatment, hospitalisation and specifically the use of ventilators, is slowed (eg until R0 = 1) and remains within the capacity of the public health system. This is about buying time, enabling capacity-building, development of effective treatments and hopefully a vaccine. In order to achieve this, social distancing is essential and contact tracing is a key mechanism.
(3) Eradication could be achieved through complete immunity of the population, but equally can be achieved through preventing the spread of the disease (R0 < 1 and eventually R0 = 0). Identification and quarantining of every single actual or potential case in the population, as well as cleansing all potential contaminated areas, are essential.

It should be noted that these can overlap and also may include “cocooning” vulnerable individuals, testing for the disease and testing subsequently for antibodies.

Each of these approaches in theory could be viable and acceptable – we use these approaches when addressing issues such as colds, flus or chicken pox. However, there are numerous issues that challenge their effectiveness and appropriateness in the context of COVID-19. Beyond issues of the rate of transmission, the potential for the virus to remain viable on surfaces or to transmit to and from animals, the potential for asymptomatic individuals to transmit the virus and the virus already being insitu within the population, there are a few other key concerns.

Firstly, the impacts of the virus are not negligible. Herd immunity is desirable, but the current mechanism to achieve it is dependent on the spread of the disease, with resulting deaths and severe impairments for members of the population. Cocooning may protect the vulnerable, but we do not know who precisely is vulnerable, and from the daily death rates in hotspots we are seeing that either cocooning is not working or the vulnerable are a much larger proportion of the population than we thought. Those pushing for flattening the curve may try to claim a moral high ground, but individuals are still badly affected by COVID-19, and for both there are knock-on effects on other treatments and the economy. On the other hand, social distancing and lockdowns can impact negatively on both the economy and mental and physical health.

Secondly, there is no certainty that immunity following recovery will arise or last long enough to prevent continued outbreaks.^21^ Due to the ongoing uncertainty, we cannot conclude that an individual cannot contract the virus a second time. If they do, it might not be as severe, but their bodies may already be physically damaged and it could lead to COVID-19’s continued circulation – necessitating continued cocooning of the vulnerable. This is relevant to all three approaches, as it undermines the herd immunity approach entirely, but also may necessitate more extended and more restrictive measures for flattening the curve and eradication – for instance, public healthcare workers treating COVID-19 patients could repeatedly contract and transmit the virus.

Thirdly, we do not know when an effective vaccine will be developed and made available in sufficient quantities. The same applies to more effective treatments. It is hoped that these will be developed rapidly, but this is by no means guaranteed, and testing will be required to ensure their safety. It might be that the only (short- or long-term) immunity possible is through contracting the disease and recovering. States may not be able to maintain social distancing or lockdowns due to practical constraints, such as a lack of resources, including finances.

Clearly, we need further information in order to know what approaches might be the most effective and acceptable in the long term. Returning to subsidiarity, though, perhaps this indicates that a decentralised approach to policy-making here is appropriate? After all, it relates to public health (varied balance of values worldwide), with little scientific certainty and where culture may play an important role.

However, even if effective, the variations raise a further significant issue that challenges individual national approaches: as we shall see, each nation state has the potential to impact negatively on the others and likewise to be impacted upon.

## IV. Global impacts: the need to adapt for pandemics?

If one returns to the three core approaches and presumes that they are each effective in principle – herd immunity will arise, case numbers and severity can be controlled and/or the disease can be eradicated within the population – simply reflect on the global nature of our society and the continued shifting nature of our populations. If we change the proportion in a population who have immunity, or introduce new sick individuals who need treatment, or simply introduce new carriers to the population, each approach will be set back and have to start again. If vulnerable individuals are no longer cocooning, they risk catching the disease and being severely impacted. Consequently, one state’s approach(es) will impact on others.^22^

The alternative? Continued controls on entry into each nation state and restrictions on travel – with entry potentially limited in future to those who have certificates of immunity (akin to yellow fever certificates) or who are willing to be quarantined for weeks. One reason why New Zealand^23^ has been able to take the eradication approach is that they started testing prior to any cases presenting symptoms and quarantined anyone entering the state. However, crucially, they are also an island distant from most other countries and entry other than by plane or ship is highly challenging – and even so, a single case in the future could lead to a new outbreak before it can be controlled, and bear in mind, for instance, the potential for unreliable tests and false negatives. Contrast this with the EU’s normally porous borders or the federal states within the USA – entry can be limited via airports and ports, but the extent of the land borders makes absolute control infeasible. Furthermore, the photos of Romanians flying to Germany and the UK to pick fruit and vegetables in the midst of a pandemic highlight the existing supply chains’ dependence on migrant workers.^24^

Similarly, the importance of a supranational or global approach, or at least considerable cooperation and collaboration, is seen in the flurry of activity and competition for resources. Most nation states are under-resourced generally, but especially are not prepared for pandemics and do not have adequate resources insitu, in part due to the economic costs and the lack of need on an ordinary basis.^25^ Nor are states typically self-sufficient in the production of protective, testing or treatment equipment or components, including ventilators or chemical reagents – we are dependent on both domestic and imported materials. That dependency is now coming to the fore, with shipments to one country being intercepted by other countries, bidding wars between States in the USA^26^ (with President Trump refusing to intervene when asked by States)^27^ and threats to block exports to other countries including Canada.^28^ Yet the stories are not all negative – countries are fast-tracking the production of necessary supplies and distributing these worldwide, including as donations, whilst others are taking in patients for treatment from overburdened countries or sending their own healthcare workers to assist.^29^ The pandemic merely highlights what we already know: the issue is not merely the limited resources, but also the control of the distribution of resources and access to them.

The benefits of centralisation for decision-making regarding policies and resource distribution are clear. They facilitate coordinated decision-making where externalities become internalised and the long-term effectiveness of the policies is facilitated. Whilst there is some centralisation internationally (with the WHO) and within the EU (with the European Centre for Disease Prevention and Control (ECDC)), this primarily involves the gathering and sharing of scientific evidence and provision of guidance.^30^ The EU does also, however, have the Health Threats Decision,^31^ which has enabled joint procurement of medical equipment and facilitated targeted distributions across the EU. Furthermore, the European Commission has taken an innovative and impressive step recently by publishing a roadmap for exiting lockdowns across the EU^32^ – thereby seeking to try and coordinate the exit strategies for the pandemic, even though the powers still rest officially with the Member States.^33^ Such centralisation is essential to address externalities and is clearly possible in principle.

However, three obvious difficulties in particular arise here. Firstly, we would need to acknowledge and address the different circumstances of each state and region. This would include the state of progression of the disease, but also the capacity limitations of states and their population to implement policies – akin to “common but differentiated responsibilities”,^34^ but leading to more proactive support internationally. Just as we put in place systems to help citizens within individual nations, there would need to be global responsibility and packages for states that did not have the means to address it. We are seeing this to an extent with the World Bank, IMF and G20’s temporary debt relief for poor countries,^35^ but more would be needed if there is to be a global approach – genuine, full-blown assistance for countries. “Public” becomes a global public. This is going to be very challenging where, for instance, there is no capacity to store food for a few weeks or to self-isolate.

Secondly, a desire for centralisation is based on the presumption that the centralised approach will be the preferable one. Whilst there is the potential to improve the effectiveness of any of the three approaches through a closely implemented and enforced approach across all populations, this does not mean it will be the best one – in light of public health objectives or otherwise. For instance, aiming for herd immunity without also effectively protecting the vulnerable and flattening the curve at the same time appears to be prioritising the economy above human health and a weaker precautionary approach. In contrast, if a complete lockdown were undertaken, this might have considerable negative impacts on society and economies if maintained for lengthy periods, but would initially seem to prioritise human health and reflect a strong precautionary approach. But which approach is preferable to society? What would happen if the “wrong” approach were taken globally, whether excessively or insufficiently precautionary?

Thirdly, linked to this, decentralisation and varied approaches provide for learning opportunities and peer pressure. Each state is looking to see what others are doing and adjusting in light of not merely new scientific data and understanding, but also in light of what seems to be working or failing.^36^ Furthermore, early, substantial restrictions and generally more precautionary approaches have influenced both the timing of responses and the underlying approach in some states (eg the UK switched from a laissez-faire, soft-regulation approach facilitating long-term herd immunity to one that is more restrictive and reflects approaches taken broadly across the EU). Consequently, there is “soft centralisation” due to comparisons facilitated by national decision-making.

Considering the importance of the values at stake and the extensive uncertainty, there is no absolute answer as to which approach will prove right in the long run or as to whether there should be full centralisation of decision-making powers (with recognition of differentiated circumstances and a relatively strong precautionary approach initially whilst information is gathered). It may be that mutual collaboration is the most feasible approach where efforts are taken to minimise externalities – but at the end of the day, unless there is a uniform approach taken globally or a vaccine is available for the entire population, the potential for one state to disrupt another’s approach will remain.

## V. Conclusion

It is necessary to consider whether public health decision-making should be elevated to the global level in the case of pandemics. Subsidiarity provides a means to consider whether such powers should be reallocated, even on a temporary basis. Both public health/human life and the economy are fundamental values within each state, and the extent of the surrounding uncertainties makes the identification of the appropriate precautionary pathway in the long term more challenging – which arguably suggests national approaches and trial and error. Nonetheless, the potential to undermine another state’s approach (externalities) and the nature and level of the potential consequences of continued circulation of the disease supports centralisation of decision-making – at least within epidemiological units – with caveats as noted.

Fundamentally, it also raises broader questions about public health, interconnectedness and values. Serious health crises exist continuously across the world, both in developed and developing countries; they affect human life and broader society, yet until the issue comes knocking at our own door, we do not step up adequately. So, yes, under ideas of subsidiarity, some centralisation of decision-making, science and distribution of resources appears logical in the context of a serious pandemic. But this leaves unanswered the question of whether some centralisation and responsibility needs to be taken outside of pandemics – of whether we need to acknowledge the relative security and privilege of some and the corresponding insecurity and vulnerability of others in both our local and global societies.

## Footnotes

1 <https://www.ecdc.europa.eu/en/geographical-distribution-2019-ncov-cases>. Accurate numbers are dependent on wide-scale testing, which is not the case.

2 IMF, “The Great Lockdown: Worst Economic Downturn Since the Great Depression”, 23 March 2020 <https://www.imf.org/en/News/Articles/2020/03/23/pr2098-imf-managing-director-statement-following-a-g20-ministerial-call-on-the-coronavirus-emergency>; IMF, “World Economic Outlook Report”, April 2020 <https://www.imf.org/en/Publications/WEO/Issues/2020/04/14/weo-april-2020>.

3 An evaluation of the normal allocation of public health powers is beyond the scope of this discussion.

4 This section draws largely on M Dobbs , “Attaining Subsidiarity-Based Multilevel Governance of Genetically Modified Cultivation?” (2016) 28(2) Journal of Environmental Law 245 .

5

L Hooghe and G Marks , “Unraveling the Central State, but How? Types of Multi-Level Governance” (2003) 97(2) American Political Science Review 233 , 233; N Chowdhury and R Wessel , “Conceptualising Multilevel Regulation in the EU: A Legal Translation of Multilevel Governance?” (2012) 18 European Law Journal 335 .

6 Eg R Vischer , “Subsidiarity as a Principle of Governance: Beyond Devolution” (2001–2002) 35 Indiana Law Review 103 , 126.

7

M Landy and S Teles , “Beyond Devolution: From Subsidiarity to Mutuality” in K Nicolaidis and R Howse (eds), The Federal Vision: Legitimacy and Levels of Governance in the United States and the European Union (Oxford, Oxford University Press 2001 ) p 414.

8

Y Blank , “Federalism, Subsidiarity, and the Role of Local Governments in an Age of Global Multilevel Governance” (2009) 37 Fordham Urban Law Journal 509 , 545–46. Reflected to an extent in Art 5(3) TEU, eg A Estella , The EU Principle of Subsidiarity and its Critique (Oxford, Oxford University Press 2002 ) p 91.

9 Dobbs, supra, note 4.

10

C Charbit , “Governance of Public Policies in Decentralised Contexts – The Multi-Level Approach” OECD Regional Development Working Papers (Paris, OECD Publishing 2011/04) pp 15–16 .

11 Eg RM Anderson , H Heesterbeek and D Klinkenberg , “How Will Country-Based Mitigation Measures Influence the Course of the COVID-19 Epidemic?” (2020) 395(10228) The Lancet 931 10.1016/S0140-6736(20)30567-5PMC715857232164834.

12 Reproduction number: the mean number of new cases an infected person will create.

13 A vague term that presumably is meant to reassure us, as the majority who die have “underlying conditions”, but without clarity to date as to what this involves – could this be a broken leg? – and also deprecating the value of those who have died who are elderly, ill or with such conditions.

14 Eg M Cascella, M Rajnik, A Cuomo, SC Dulebohn and R Di Napoli, “Features, Evaluation and Treatment Coronavirus (COVID-19)”, 6 April 2020 <https://www.ncbi.nlm.nih.gov/books/NBK554776>.

15 Eg L Rosenbaum, “Facing Covid-19 in Italy – Ethics, Logistics and Therapeutics on the Epidemic’s Front Line”, *The New England Journal of Medicine*, 18 March 2020 <https://www.nejm.org/doi/full/10.1056/NEJMp2005492>; TP Hanna , GA Evans and CM Booth , “Cancer, COVID-19 and the Precautionary Principle: Prioritizing Treatment During a Global Pandemic” (2020) 17 Nature Reviews Clinical Oncology 268 10.1038/s41571-020-0362-6PMC711755432242095.

16

L Petetin , “The COVID-19 Crisis: An Opportunity to Integrate Food Democracy into Post-Pandemic Food Systems” (2020) European Journal of Risk Regulation , forthcoming.

17 Anderson et al, supra, note 11.

18 Eg in the UK initially: S Boseley, “New Data, New Policy: Why UK’s Coronavirus Strategy Changed”, *The Guardian*, 16 March 2020 <https://www.theguardian.com/world/2020/mar/16/new-data-new-policy-why-uks-coronavirus-strategy-has-changed>.

19 Eg a common approach, including most of the EU: CORDIS, “Trending Science: Why Has ‘Flatten the Curve’ Become the Public Health Mantra in the Global Fight against Coronavirus?” <https://cordis.europa.eu/article/id/415751-flatten-curve>.

20 Eg in New Zealand, following an initial flattening-the-curve approach: M Brockett, “This Country Says It’s on Course to Wipe Out Covid-19”, *Bloomberg News*, 16 April 2020 <https://www.bloomberg.com/news/articles/2020-04-16/new-zealand-seeks-to-wipe-out-virus-after-early-lockdown-success>.

21 K O’Sullivan, “No Proof of Immunity in Recovered Coronavirus Patients, Says WHO”, *The Irish Times*, 18April 2020 <https://www.irishtimes.com/news/health/no-proof-of-immunity-in-recovered-coronavirus-patients-says-who-1.4232563>.

22 G Scally, “North and Republic Must Harmonise Covid-19 Response”, *The Irish Times*, 31 March 2020 <https://www.irishtimes.com/opinion/gabriel-scally-north-and-republic-must-harmonise-covid-19-response-1.4216073>.

23 Brockett, supra, note 20.

24 L O’Carroll, “Romanian Fruit Pickers Flown to UK amid Crisis in Farming Sector”, *The Guardian*, 15 April 2020 <https://www.theguardian.com/world/2020/apr/15/romanian-fruit-pickers-flown-uk-crisis-farming-sector-coronavirus>.

25 The main obvious exception being Cuba, which has been providing considerable assistance to other states.

26 D Smith, “New York’s Andrew Cuomo Decries ‘EBay’-Style Bidding War for Ventilators”, *The Guardian*, 31 March 2020 <https://www.theguardian.com/us-news/2020/mar/31/new-york-andrew-cuomo-coronavirus-ventilators>.

27 Q Forgey, “‘We’re Not a Shipping Clerk’: Trump Tells Governors to Step up Efforts to Get Medical Supplies”, *Politico*, 19 March 2020 <https://www.politico.com/news/2020/03/19/trump-governors-coronavirus-medical-supplies-137658>.

28 H Jackson, “Coronavirus: Canada to Receive ‘millions’ of Masks from China, Trudeau Says”, *Global News*, 4 April 2020 <https://globalnews.ca/news/6778318/coronavirus-trudeau-aid-vulnerable>.

29 Eg J Bateman, “‘Solidarity Knows No Borders’: Germany Treating Dozens of Coronavirus Patients from Italy and France”, *The Independent*, 1 April 2020 <https://www.independent.co.uk/news/world/europe/coronavirus-germany-italy-france-hospital-treatment-covid-19-a9440906.html>; NG Torres and J Charles, “Despite US Warnings, Cuba’s Medical Diplomacy Triumphs in the Caribbean during Pandemic”, *Miami Herald*, 15 April 2020 <https://www.miamiherald.com/news/nation-world/world/americas/cuba/article241745281.html>.

30 The WHO’s Pandemic Influenza Pandemic Framework, which was created by non-binding resolution, is of particular interest. Although not applicable to COVID-19, it demonstrates the potential role of the WHO in encouraging and facilitating sharing of research and resources – but equally through its uniqueness demonstrates the challenges in creating even soft instruments at an international level, much less legally enforceable ones.

31 Decision No 1082/2013/EU of the European Parliament and of the Council of 22 October 2013 on serious cross-border threats to health, [2013] OJ L293/1 – created post-SARS and H1N1.

32 European Commission, “A European Roadmap to Lifting Coronavirus Containment Measures” (Communication 15 April 2020) <https://ec.europa.eu/info/live-work-travel-eu/health/coronavirus-response/european-roadmap-lifting-coronavirus-containment-measures_en>.

33 For further on the European approach, see AM Pacces and M Weimer , “From Diversity to Coordination: A European Approach to COVID-19” (2020) European Journal of Risk Regulation , forthcoming; A Renda and RJ Castro , “Towards Stronger EU Governance of Health Threats after the COVID-19 Pandemic” (2020) European Journal of Risk Regulation , forthcoming.

34 Eg Principle 7 of the Rio Declaration 1992, or Arts 3 and 4 of the UN Framework Convention on Climate Change.

35 France24, “Covid-19: G20 Endorses Temporary Debt Relief for the Poorest Countries”, 15 April 2020 <https://www.france24.com/en/20200415-covid-19-g20-endorses-temporary-debt-standstill-for-the-poorest-countries>.

36 Eg even whilst coordinating with surrounding American States, New York City Governor Cuomo said, “Remember none of this has been done before … I want to learn from those other countries frankly … what worked and what didn’t work.” As reported in: S Lynch, “Trump Clashes with Reporters as He Defends Coronavirus Record”, *The Irish Times*, 14 April 2020 <https://www.irishtimes.com/news/world/us/trump-clashes-with-reporters-as-he-defends-coronavirus-record-1.4228432>.

